# Electronic capturing of patient-reported outcome measures on a touchscreen computer in clinical diabetes practice (the DiaPROM trial): a feasibility study

**DOI:** 10.1186/s40814-019-0419-4

**Published:** 2019-02-20

**Authors:** Ingvild Hernar, Marit Graue, David Richards, Ragnhild B. Strandberg, Roy M. Nilsen, Grethe S. Tell, Anne Haugstvedt

**Affiliations:** 1 Department of Health and Caring Sciences, Western Norway University of Applied Sciences, P.O. Box 7030, N-5020 Bergen, Norway; 2 Department of Global Public Health and Primary Care, University of Bergen, Bergen, Norway; 3 Department of Medicine, Haukeland University Hospital, Bergen, Norway; 4 Institute for Health Research, University of Exeter Medical School, Exeter, UK; 5 Department of Research and Development, Haukeland University Hospital, Bergen, Norway

**Keywords:** Patient-reported outcome measures, Electronic data collection, Feasibility, Diabetes practice, Type 1 diabetes, Routine assessment, Diabetes-related distress, Psychological well-being

## Abstract

**Background:**

Living with type 1 diabetes (T1D) is demanding, and emotional problems may impair ability for diabetes self-management. Thus, diabetes guidelines recommend regular assessment of such problems. Using patient-reported outcome measures (PROMs) to assess diabetes-related distress and psychological well-being is considered useful. It has been proposed that future work should examine the use of PROMs to support the care of individual patients and improve the quality of health services. To our knowledge, the use of PROMs has not been systematically evaluated in diabetes care services in Norway. Electronically captured PROMs can be directly incorporated into electronic patient records. Thus, the study’s overall aim was to examine the feasibility and acceptability of capturing PROMs electronically on a touchscreen computer in clinical diabetes practice.

**Methods:**

Adults with T1D age ≥ 40 years completed PROMs on a touchscreen computer at Haukeland University Hospital’s diabetes outpatient clinic. We included 46 items related to diabetes-related distress, self-perceived diabetes competence, awareness of hypoglycaemia, occurrence of hyperglycaemia, hypoglycaemia and fluctuating glucose levels, routines for glucose monitoring, general well-being and health-related quality of life. Participants subsequently completed a paper-based questionnaire regarding comprehension and relevance of the PROMs, acceptance of the number of items and willingness to complete electronic PROMs annually. We wrote field notes in the outpatient clinic based on observations and comments from the invited participants.

**Results:**

During spring 2017, 69 participants (50.7% men), age 40 to 74 years, were recruited. Generally, the touchscreen computer functioned well technically. Median time spent completing the PROMs was 8 min 19 s. Twenty-nine (42.0%) participants completed the PROMs without missing items, with an 81.4% average instrument completion rate. Participants reported that the PROMs were comprehensible (*n* = 62) and relevant (*n* = 46) to a large or very large degree, with an acceptable number of items (*n* = 51). Moreover, 54 were willing to complete PROMs annually. Participants commented that the focus on living with diabetes was valued.

**Conclusions:**

Capturing PROMs on a touchscreen computer in an outpatient clinic was technically and practically feasible. The participants found the PROMs to be relevant and acceptable with a manageable number of items, and reported willingness to complete PROMs annually.

**Electronic supplementary material:**

The online version of this article (10.1186/s40814-019-0419-4) contains supplementary material, which is available to authorized users.

## Background

Living with type 1 diabetes (T1D) is demanding. The condition requires lifelong insulin therapy and constant attention to complex self-management tasks. Among adults with T1D, more than half do not reach recommended treatment goals for glycaemic control [1–3]. Although this could be explained by improper treatment regimen, psychological and psychosocial aspects may be significant barriers for diabetes self-management and glycaemic control [4–6]. Consequently, several diabetes guidelines recommend regular assessment of psychological well-being and diabetes-related distress in people with diabetes [7–9]. Although essential in recognition of individual needs [10, 11], psychological and psychosocial aspects are greatly underreported in clinical care [6, 12, 13].

Patient-reported outcome measures (PROMs) have been developed to assess patients’ perceptions of living with a condition and its impact on health status, health-related quality of life and/or other health-related constructs [14, 15]. PROMs are used in clinical trials to assess the effect of interventions on health-related outcomes, but are also useful in enabling patients with chronic conditions to raise or share concerns with healthcare providers in clinical consultations [16]. PROMs are typically self-administered and can be administered on paper or by electronic devices, either in the patient’s home or at the clinic [17–19]. Transferring paper-based instruments to electronic interfaces may produce data with psychometric equivalence as long as substantive content alterations are not made [18, 20, 21].

Compared to paper-based PROMs, electronic systems have potential benefits such as reducing missing and unusable data by not allowing people to continue registration without completing all items, and only allowing one response option per item [19, 22]. Some claim scoring on paper is more time consuming compared to electronic scoring [23]. While the logistics of entering paper data into the electronic patient records (EPR) raise questions regarding responsibility for the data entry, electronically captured PROMs can be directly incorporated into the EPR resulting in less administrative burden [16, 19, 22]. In recent years, the use of self-report instruments to monitor quality of care has increased, with data also being fed into medical quality registers [16]. It has been proposed that future work should examine the use of PROMs to support the care of individual patients and at the same time improve the quality of health services [14, 24].

To our knowledge, the use of PROMs has not been systematically evaluated in diabetes care services in Norway. In accordance with the UK Medical Research Council’s framework for researching complex interventions [25, 26], we have therefore designed the DiaPROM trial (ClinicalTrials.gov ID: NCT03471104) for people with type 1 diabetes, where electronically captured PROMs will be used to identify individual needs and promote goal-oriented clinical diabetes consultations. The findings of the present feasibility study will inform a pilot randomised controlled trial (RCT).

## Methods

### Aim

The overall aim of the present study was to examine the feasibility and acceptability of capturing PROMs electronically on a touchscreen computer in clinical diabetes practice.

Our specific objectives were:

1. To evaluate our proposed recruitment strategy by estimating the proportion of eligible participants who consent to participate.
2. To examine the feasibility of the technical and practical procedures for collecting PROMs on a touchscreen computer in the outpatient clinic.
3. To assess the participants’ perceptions about the PROMs used, including their comprehension of items, acceptability of number of items, relevance of items and willingness to complete electronic PROMs at their future annual clinical consultations.

### Design

We undertook an uncontrolled feasibility study using cross-sectional data and field observations to examine crucial elements of a subsequent pilot RCT.

### Setting and participants

The study was conducted at Haukeland University Hospital in Western Norway covering about one million inhabitants including both rural and urban areas. We recruited participants with T1D aged ≥ 40 years during 6 weeks from April to June 2017. The reason for choosing this age group was to not include potential participants for the coming pilot RCT, which is planned for young adults < 40 years [27]. We identified eligible participants from the endocrinology outpatient clinic’s planned consultations. Approximately 1 week prior to the consultations, administrative staff sent a written information and consent form by postal mail inviting eligible participants to take part in the study. We asked the patients to come to the hospital at least 10 min before the scheduled consultation. People who were unable to read or complete the PROMs on the touchscreen computer were excluded. Furthermore, we did not invite patients with the following conditions recorded in their medical records: cognitive deficiency (e.g. Down’s syndrome, Alzheimer), severe medical comorbidity (e.g. end-stage renal disease, severe heart failure, severe cancer), and/or a major psychiatric diagnosis (e.g. severe depression or bipolar disorder, schizophrenia) as the burden to complete PROMs might be too challenging.

### Data collection

#### Sample characteristics

We collected the following sociodemographic and diabetes-related information from the participants’ EPR: age, sex, ethnicity, diabetes type, diabetes duration, diabetes long-term complications, glycosylated haemoglobin (HbA_1c_) level and insulin injection device. We also obtained self-report data on first language, current educational level, marital/cohabitation status and work affiliation. In addition, the ethical committee permitted us to register age and sex of those who declined participation, using the EPR’s patient administration system.

#### Recruitment

We recorded the number of eligible participants who were invited to participate, number of people who attended consultations, and number of people who agreed to participate. In addition, we observed whether eligible participants approached the touchscreen computer by themselves or if they needed a reminder from a project member (IH, RBS or AH), who were present in the waiting area during the recruitment period.

#### Technical and practical procedures for collecting PROMs

The touchscreen computer (17″ screen) was placed inside a metal cabinet (kiosk). We gathered data on the technical and practical performance of the computer and observed participants’ ability to complete the PROMs. The leading supplier of eHealth systems to Norwegian hospitals, DIPS AS [28], developed the technical application which included the software for completing the PROMs, a secure data repository for temporary PROMs data storage and the method for transferring the PROMs data to the participants’ diabetes-specific health records. This diabetes-specific record is also the Norwegian Diabetes Register for Adults’ electronic tool for collecting register data from outpatient clinics [29]. We used the hospital’s wireless local area network (WLAN) and a USB dongle to boost connectivity. The kiosk was situated next to the outpatient clinic’s waiting area to ensure visibility. “Questions for people with diabetes” was displayed on the screen and a poster with information was placed next to the screen. By tapping the screen, information concerning the data collection procedure and the measured constructs were displayed, and the PROMs appeared one item at a time. Respondents could either tap “next” or wait 2 s for the computer to automatically continue to the next item. In addition, respondents could also tap “back” to review or change their previous responses. On the top of the screen, a row of small boxes signalled how many of the items were responded to and the number left to complete.

The software utilised time stamps to track time needed (minutes and seconds) for completing the PROMs. Participants were not required to log in using personal identification; instead, the application generated a four-character code with a mix of letters (A–Z, except I and O) and numbers (1–9) for each session. Participants were instructed to write down their unique code on a paper form placed next to the computer and to bring this form to the consultation. The code was then used to download the PROM data from the secure data repository to the diabetes-specific records.

#### Instruments and participants’ perceptions about the PROMs

We used the Problem Areas in Diabetes scale (PAID) to assess diabetes-related distress related to living with diabetes and its treatment [30–32]. This instrument is considered appropriate in achieving therapeutic and goal-oriented consultations [33, 34]. We used the Perceived Competence for Diabetes Scale (PCDS) to map self-perceived ability for diabetes self-management [35, 36], and “The Gold” scale to assess awareness of hypoglycaemia [37]. In addition, we developed three questions asking the participants to assess self-perceived occurrence of hyperglycaemia, hypoglycaemia and fluctuating glucose levels over the latest couple of weeks. Furthermore, we included the World Health Organization 5-Well-Being Index (WHO-5), a generic measure for psychological well-being [38, 39], and the RAND-12 Health Status Inventory (RAND-12) to assess health-related quality of life [40, 41]. Finally, we added items related to the use of glucose monitoring devices and frequency of glucose measurements. In total, 47 items were included in the questionnaire. A description of the included PROMs is shown in Additional file 1*.* Acceptable psychometric properties have been reported for the PAID [42], the PCDS [43], “the Gold” [37], the WHO-5 [39], and the RAND-12 [40]. Cronbach alphas in the present study were PAID 0.94, PCDS 0.94, WHO-5 0.84, and RAND-12 0.89.

The PROMs were originally developed for paper-based administration, with an introductory sentence preceding all items. In our electronic versions, one item appeared at a time, thus the introductory sentences were adapted and placed directly above all items to avoid respondents having to scroll back and forth to read this information. We did not alter the wording of any items or response options. However, for the response options to fit the screen, we had to alter the layout from horizontal to vertical positioning for all instruments, except “the Gold”. In addition, we added “unanswered” as the default response option for all items, allowing participants to skip a question and proceed to the next one, and it was only possible to choose one response option per item.

The participants also responded to a paper-based questionnaire concerning their perceptions about the PROMs. The questionnaire comprised questions regarding comprehension, perceived relevance, and acceptance of the number of items included in the PROMs. Finally, we asked about participants’ willingness to complete electronic PROMs annually. We looked to the Norwegian Institute of Public Health’s user experience questionnaires for item wording and response alternatives [44]. Finally, we added space for individual written feedback and encouraged the participants to comment on the procedures, the included items and scales in their own words. In addition, the project member present in the clinic was available if any of the participants preferred to share opinions verbally. We wrote field notes based on observations and comments from participants and those who were invited to participate but declined.

### Analysis

We used Stata SE 15 for Windows for all statistical analyses [45]. We applied descriptive statistics for demographic characteristics. In order to estimate the proportion of participants who would meet the inclusion criteria for the planned pilot RCT [46], we calculated the proportion of participants with single-item PAID scores ≥ 3 or total scores ≥ 30. Prior to analyses, we substituted missing PAID items by participants’ mean score if minimum 18 (of 20) items were completed [47, 48]. Furthermore, we examined differences between male and female participants regarding total PAID scores, PAID ≥ 30, PAID ≥ 40 and item scores ≥ 3.

In order to evaluate the recruitment strategy, we registered the number of invited participants. Then we calculated the number and percentage of people who attended consultations and number and percentage of people who agreed to participate. We quantified the proportion of missing items (frequencies and percentages) and calculated the duration of the PROM sessions (median, minimum and maximum). In addition, we quantified the variables concerning comprehension of the PROMs, acceptability of number of items, relevance of PROMs and willingness for annual completion of electronic PROMs using frequencies and percentages.

Finally, we organised the field notes concerning our observations of technical and practical aspects and participants’ comments chronologically by the date these were collected. Two of the researchers (IH and RBS) independently read the document and summarised the content describing the activities that took place in the waiting area. The text was adjusted and agreed by the project members who had been present in the waiting area.

## Results

### Recruitment

**Fig. 1:**
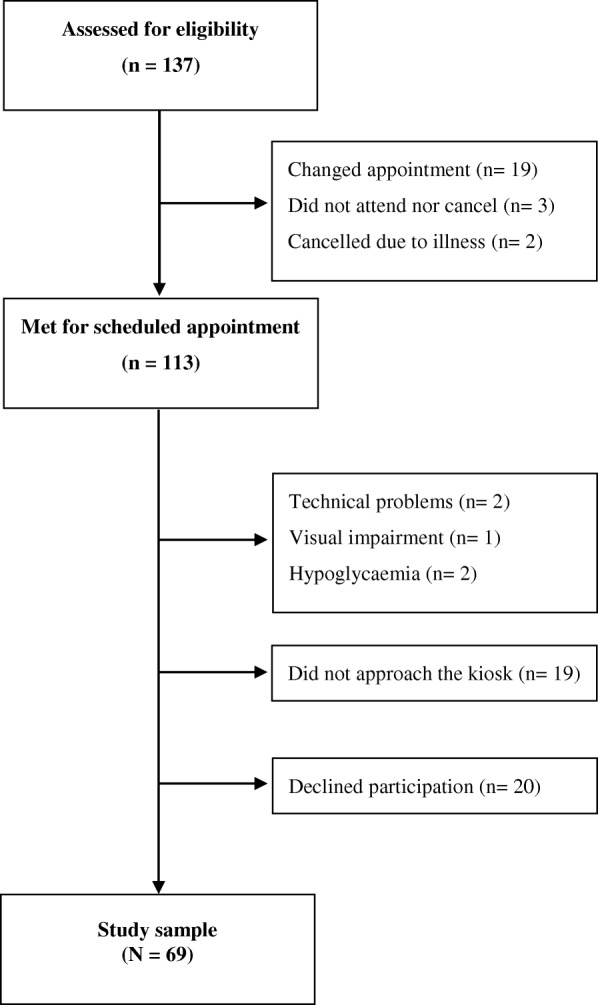
The recruitment and inclusion of adults with type 1 diabetes in a Western Norway university hospital outpatient clinic. The DiaPROM trial feasibility study

We invited 137 adults with T1D (72 men, 65 women) of whom 24 (17.5%) did not attend their scheduled outpatient clinic consultations (median age 47 yrs. (41–71), 58.3% men), leaving 113 potential participants (51.3% men) (Fig. 1). Five eligible participants (2 men, 3 women) did not participate due to technical (*n* = 2) or medical (*n* = 3) issues, and 20 (17.7%) declined participation (median age 48 yrs. (40–71), 55% men). On occasions where project members were not available for guidance at the outpatient clinic, 19 (out of 32) eligible participants did not approach the kiosk and thus did not participate (median age 48 years (41–59), 52.6% men). Finally, 69 (61.1%) participants (35 men, 34 women) completed the PROMs on the touchscreen computer. Most of the invited participants had to be reminded about the invitation and shown the location of the kiosk. Therefore, we included a picture of the kiosk in the information letter halfway through the recruitment period, which appeared to lead to more participants finding the kiosk by themselves.

Characteristics of the participants are presented in Table 1. All but three reported Norwegian as their first language. Over one half reported having university or college education, and 27 (41.5%) were in full-time employment. Women were slightly older than men, but men had longer diabetes duration (31 vs. 19 years). The median HbA1c value was 60.7 mmol/mol (7.7%). Thirty-five (50%) participants had at least one diabetes long-term complication, and retinopathy was the most frequent complication seen in 28 (40.6%). We found that nearly half of the participants met the inclusion criteria for the planned pilot RCT, and that two thirds of these were women (Table 2).

**Table 1:** Demographic characteristics among adults with type 1 diabetes attending an outpatient clinic in a Western Norway university hospital

Total	*N* = 69
Male sex, *n* (%)	35 (50.7)
Age (years) (median, min-max)	51.0 (40–74)
First language, *n* (%) ^2^	
Norwegian	62 (95.4)
Other Scandinavian language	1 (1.5)
Other European language	2 (3.1)
Educational level, *n* (%) ^3^	
Primary school	5 (7.8)
Secondary school	25 (39.1)
University/college ≤ 4 years	17 (26.55)
University/college > 4 years	17 (26.55)
Work affiliation, *n* (%) ^2^	
Full-time work	27 (41.5)
Part-time work	9 (13.9)
Unpaid work	2 (3.1)
Unemployed	2 (3.1)
On sick leave/benefits	16 (24.6)
Retired	6 (9.2)
Other/not specified	3 (4.6)
Living alone, *n* (%) ^2^	9 (13.9)
Diabetes duration (years) (median, min-max)	26.0 (1–67)
HbA_1c_ (mmol/mol) (median, min-max)	60.7 (41.0–107.7)
HbA_1c_ (%) (median, min-max)	7.7 (5.9–12.0)
At least one long-term complication, *n* (%)	35 (50.7)
Insulin injection device, *n* (%)	
Pen	43 (62.3)
Pump	26 (37.7)
Glucose monitoring device, *n* (%) ^1^	
SBGM	47 (71.2)
FGM	3 (4.6)
CGM	16 (24.2)

*HbA*_*1c*_ haemoglobin A_1c_, *SBGM* self-blood glucose monitoring, *FGM* flash glucose monitoring, *CGM* continuous glucose monitoring

^1^*n* = 66, ^2^*n* = 65, ^3^*n* = 64 due to missing data

**Table 2:** PAID scores in adults with type 1 diabetes, including the proportion eligible for extra follow-up according to the planned intervention inclusion criteria. The DiaPROM trial feasibility study

	Total (*N* = 69)	Men (*n* = 35)	Women (*n* = 34)
PAID score (0–100) ^1^			
Median (min-max)	22.4 (1.3–65.0)	21.3 (1.3–58.8)	32.5 (2.5–65.0)
Mean (SD)	25.9 (16.2)	21.4 (13.8)	31.1 (17.3)
PAID score ≥ 30, *n* (%) ^1^	26 (39.4)	9 (25.7)	17 (50.0)
PAID score ≥ 40, *n* (%) ^1^	11 (16.7)	3 (8.6)	8 (25.8)
Minimum one PAID item ≥ 3, *n* (%)	28 (40.6)	11 (31.4)	17 (50.0)
^#^PAID score ≥ 30 and/or minimum one item scored ≥ 3, *n* (%)	34 (49.3)	12 (34.3)	22 (64.7)

^#^The planned intervention inclusion criteria for the DiaPROM trial are a total score ≥ 30 or single-item PAID scores ≥ 3

^1^*n* = 66 due to missing data (3 women)

### Technical and practical procedures for collecting PROMs

**Fig. 2:**
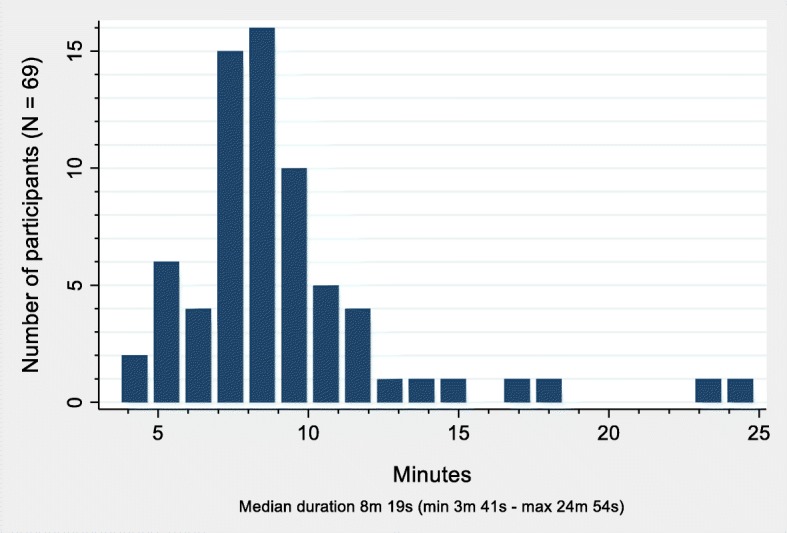
Time needed to complete an electronic questionnaire on a touchscreen computer by adults with type 1 diabetes in an outpatient clinic at a Western Norway university hospital. The DiaPROM trial feasibility study

The touchscreen computer mostly functioned well. However, we noticed that PROM sessions had been started but not finished on several occasions, which meant that sometimes a participant who was to start a new session found parts of the PROMs displayed instead of the start screen. As a result, technicians from DIPS programmed the application to display a 1 min inactivity notification with a 15-s countdown, and to stop the session if the screen was not touched during the countdown. Participants’ median duration (minutes and seconds) for completing PROMs was 8 min 19 s (min 3 min 41 s–max 24 min 54 s) (Fig. 2). One man and one woman used > 20 min.

Comments expressed by the participants and logged in the field notes, indicated that participants in general expressed a positive attitude towards completing PROMs in the waiting area, favouring this option compared to an internet-based solution (e.g. from home). However, limited time spent in the waiting area ahead of the consultation was stated as a motive for wanting to complete PROMs at home in the future. Some participants found the two methods for proceeding to the next item confusing and suggested that it should be either automatic or touch-based. Regarding the four-character code, some handwritten letters and numbers were difficult to interpret (e.g. A and 4, B and 8, G and 6 and also Z and 2). Consequently, we will avoid these letters in the pilot RCT.

### Participants’ perceptions about the PROMs

**Fig. 3:**
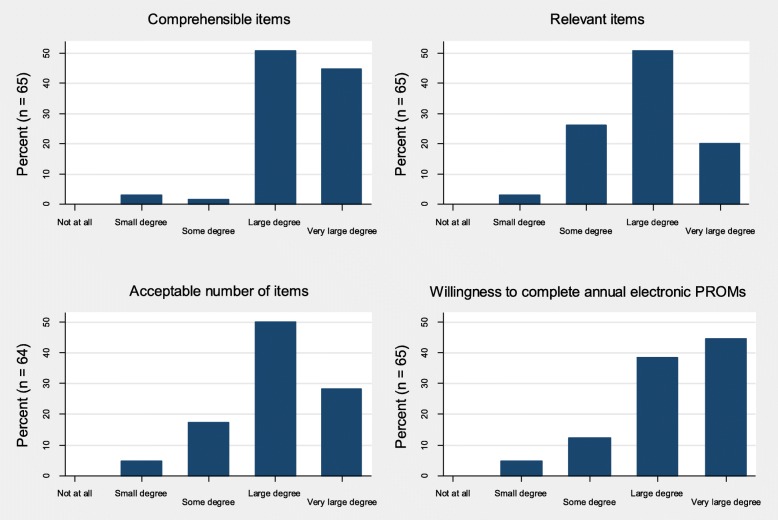
Adults with type 1 diabetes and their perceptions about completing an electronic questionnaire on a touchscreen computer in an outpatient clinic at a Western Norway university hospital. The DiaPROM trial feasibility study

Of the 69 participants, 65 completed the paper questionnaire regarding their perceptions about the PROMs. The PROM items were reported to be comprehensible to a large or very large degree by 62 (95.4%) participants, and 46 (70.8%) found the PROMs relevant at least to a large degree (Fig. 3). Fifty-one (78.1%) participants reported that the number of items was acceptable to a large or very large degree, and 54 (83.1%) reported willingness to complete PROMs annually at least to a large degree. Twenty-nine (42.0%) participants completed all PROMs without any missing items, 13 (18.8%) had one missing item, 12 (17.4%) had two missing items and the remaining 15 (21.2%) had three to 12 missing items (Table 3). The instruments’ completion rates varied from 72.5 to 91.3% (Table 3) with an average rate of 81.4%.

**Table 3:** Number of individuals, *n* (%) with missing PROMs items among adults with type 1 diabetes attending an outpatient clinic in a Western Norway university hospital. The DiaPROM trial feasibility study

Self-report instruments	No. items	0 missing	1 missing	2 missing	3 missing	4 missing	5 missing
The WHO 5-Well-Being Index (WHO-5)	5	50 (72.5)	16 (23.2)	3 (4.3)	0 (0.0)	0 (0.0)	0 (0.0)
The Problem Areas in Diabetes scale (PAID) ^1^	20	53 (76.8)	10 (14.5)	3 (4.35)	1 (1.45)	0 (0.0)	1 (1.45)
Perceived Competence in Diabetes Scale (PCDS)	4	59 (85.5)	8 (11.6)	1 (1.45)	0 (0.0)	1 (1.45)	–
Perceived elevated, low and varied blood glucose values	3	59 (85.5)	5 (7.25)	0 (0.0)	5 (7.25)	–	–
Awareness of hypoglycaemia (“Gold”)	1	63 (91.3)	6 (8.7)	–	–	–	–
RAND-12 Health Status Inventory (RAND-12) ^2^	12	53 (76.8)	9 (13.0)	3 (4.35)	1 (1.45)	2 (2.9)	0 (0.0)

*PROMs* patient-reported outcome measures

^1^ One person had seven missing PAID items. ^2^ One person did not complete the RAND-12 (*n* = 68)

In the field notes, we found that the majority of participants who commented verbally on the PROMs’ contents found them relevant and valued the focus on experiences of living with diabetes. However, participants interpreted the question concerning relevance of items differently. Some regarded it as being relevant to them personally at that point in time, while the question was intended to ask about general relevance for people with diabetes. Some questions were reported by participants as somewhat overlapping, but it was noted by other participants that some overlap could yield more nuanced information.

## Discussion

In this study, we found that using a touchscreen computer for capturing PROMs electronically in our outpatient clinic setting was technically and practically feasible. The majority of participants reported the included PROMs to be acceptable and relevant. One half of the participants had either a PAID score ≥ 30 or a minimum one item scored ≥ 3, which indicated diabetes-related distress of concern, and participants with such scores qualify for extra follow-up in the upcoming pilot RCT. The mean PAID scores were in line with other studies of similar patient groups [43, 49]. Nearly two thirds of participants with elevated diabetes-related distress scores were women. Others have reported similar sex differences, especially in younger adults with T1D [50, 51].

### Recruitment strategy

The recruitment of the 69 participants took 6 weeks. Keeping track of eligible participants who changed or did not keep their appointments was demanding. We observed that only a handful of invited participants who attended the clinic seemed to have considered participation prior to coming to the hospital, and the majority did not approach the touchscreen computer by themselves. After we included a picture of the kiosk in the information letter, more participants approached it without guidance. Nonetheless, efficient recruitment appeared to depend on the presence of a person who could provide information, support participants and manage the recruitment logistics, a phenomenon also identified by Treweek [52]. Establishing new routines is in general challenging and will often require extra resources, especially in the earlier phases of implementation initiatives. Those who arrived shortly before the consultation did not have time to complete the PROMs and could therefore not participate unless the healthcare personnel was delayed. This may indicate that our recommendation of coming to the clinic at least 10 min ahead of the appointment was not adequately emphasised in the information letter. Preparing written study information requires the researchers to carefully consider wording and amount of text. The ethics committees’ demands for compulsory text makes this task even more demanding. Thus, user involvement in preparing information is of utmost importance.

### Technical and practical procedures for collecting PROMs

Electronic capturing of PROMs using a touchscreen computer was the only administration method offered in this feasibility study. Although we found no indication that the data collection method represented an obstacle for participation, it may have influenced recruitment due to perceived technology barriers or the location of the kiosk. Recent meta-analyses and reviews refer to mixed results on preferences for electronic versus paper-based administration, ranging from 50% [18] to 87% [53] in favour of the electronic format. This suggests that patients of all ages have become increasingly more familiar with electronic devices, and using multiple methods for collecting PROMs and allowing multiple places for completing them might improve response rates [54]. However, the general recommendation is to avoid mixing modes within a study [22], since different administration methods require somewhat different skills and resources of those completing the PROMs [23].

We had to perform some minor layout changes when we adapted the paper-based PROMs to the electronic interface, but this was done in accordance with recommendations supporting equivalence of paper- and computer-administered PROMs [19–21]. However, the visual look thus turned out to differ a bit as multiple items are generally presented on the same page in paper-based PROMs, whereas electronic formats present one item at a time [20, 22]. We used a relatively large screen (17″), but still it was not possible to retain all items and response options of each self-report instrument on the same screen without compromising the font size. Hence, we chose the single item per screen approach to provide consistency across all instruments [22]. This also meant that we could present the items with relatively large fonts, making it more accessible to people with minor visual impairments.

We chose to locate the kiosk in close proximity to the outpatient clinic’s waiting area to make it visible and easy to access, but at the same time not too close to the seating area for privacy reasons [23]. We received no negative comments on the location, neither about how the PROM items were presented on the screen. However, 19 out of 32 eligible participants did not approach the kiosk when the project members were not available for guidance. Furthermore, we registered that a number of PROM sessions had been started but not finished. This could be a result of questions being presented one at a time and the total number of items appearing to be too many for some people. In addition, people not eligible for the study might have been curious about the screen and its contents and thus might have started a PROM session without finishing. According to recommendations [55], completing PROMs in a clinical setting should not take more than 12–15 min. In our study, the median session duration was less than 9 min. Nonetheless, 16 participants (23.1%) used more than 10 min and 4 (5.7%) used more than 15 min. Hence, in similar studies, participants completing a questionnaire of 47 items should be encouraged to come to the clinic at least 15 min before their consultation.

We experienced few technical and practical problems during the study. WLAN connectivity problems could have been avoided using a cabled network. Due to possible misinterpretation of handwriting, we considered using printers for delivering the four-character code on slips of paper, but this could entail other logistical and technical issues, plus extra costs. Other in-clinic PROM studies report involving clinicians for logging the respondents into the electronic solution [56]. We did not develop this option as the outpatient clinic leaders were clear that it would not be possible to allocate personnel for this task in the future. In addition, we chose to avoid personal identification solutions such as BankID, a Norwegian cloud infrastructure allowing electronic ID, authentication and signing [57]. Due to the application’s integration with the EPR, this would involve greater system security needs. Using the personal codes as described, the participants were in charge and control of their codes, available for interpretation, and we avoided security risks.

### Participants’ perceptions about the included PROMs

We chose a mix of generic and diabetes-specific instruments, which could have affected the perceived relevance of the PROMs. However, combining generic and condition-specific PROMs may result in a more in-depth assessment of health-related outcomes [23]. Although generic measures might not be considered relevant in follow-up of diabetes, condition-specific instruments may miss other health-related dimensions possibly unrelated to the condition, but still affecting patients [17, 58]. Several participants’ expressed appreciation of the focus on psychosocial aspects of living with diabetes. Using PROMs to capture the participants’ perceptions of their own health and thereby informing clinical practice thus has the potential to facilitate increased person-centred care [14, 17, 55].

The average PROM completion rate of 81.4% was relatively high. Therefore, our method for electronic capturing of data seems adequate. In addition, it might also reflect that the number and relevance of the questions were acceptable. Some argue that electronic PROM systems can lead to more complete and accurate datasets due to a reduction of missing or unusable data [16, 17, 19]. The method ensures that out-of-range, contradictory and/or extraneous responses are not possible. Furthermore, data entry errors are minimised since manual punching is not needed [17, 19, 23]. Although computer technologies require investment in software and hardware, collecting PROMs electronically is regarded as more economical concerning time and personnel resources compared to traditional paper-based collection [23, 59]. However, 40 (58.0%) participants did not respond all items, where 25 (37.9%) completed all but one or two items. Except for one case, the missing data were due to incomplete instrument sections. The results are similar to another recent feasibility study reporting on collecting electronic PROMs (33 items), where 47.1% of the participants completed all items [56]. Lack of complete datasets is one of the greatest practical challenges related to the use of PROMs. Unfortunately, there is no generally accepted standard approach for handling missing PROMs data, and preventing missing data with a design that supports PROM completion is probably the most effective solution [60].

### Strengths and limitations

We consider it a strength that the study included both men and women with long diabetes duration and experience with attending outpatient clinic consultations. Moreover, we collaborated with healthcare professionals with highly specialised information technology (IT) competence who had the skills to make necessary and timely improvements of the touchscreen application. Furthermore, it is a strength that we incorporated healthcare user involvement from the beginning of the design and development of the study in accordance with the GRIPP2 short form [61].

A relatively small, homogenous Norwegian sample limits generalisability. The findings were analysed descriptively due to the small sample and cross-sectional design. Recruitment was challenging since most participants had to be reminded about the study invitation and therefore did not approach the kiosk by themselves. Non-response is always a concern in recruitment and data collection since non-responders may be systematically different from those providing complete data [62], and the distribution of missing data across a range of measures also suggests this. Since we only used an electronic method for collecting PROMs, participation was limited to individuals capable of and interested in using the touchscreen computer. Therefore, participation may have been biased towards educated and younger informants. Our sample’s educational level was higher than the Norwegian average for 40- to 67-year-olds, where 35% have university or college education and 22% have primary school only [63]. Consequently, our results may be limited to those familiar with electronic devices. Notwithstanding that, the public is becoming more experienced with using IT [53]. According to the 2018 Digital Economy and Society Index, 77% of Norwegian people have basic digital skills at least, and 96% are internet users [64], indicating capability of using a computer. By excluding groups of people unable to complete the electronic PROMs, we might lack potentially valuable insight regarding the impact of diabetes on these people’s lives. For this group, completing PROMs with assistance could be an option. At this point, we chose to focus on an electronic data collection method. Finally, we consider it a limitation that the healthcare service users were not involved in preparing the written information for the present study.

## Conclusions

We found that capturing PROMs on a touchscreen computer in the waiting area in connection with attending an outpatient clinic consultation was technically and practically feasible, and we identified only minor technical issues that will be improved prior to the pilot study. The majority of participants found the PROMs relevant and acceptable with a manageable number of items, and reported willingness to complete electronic PROMs annually in the future.

## Additional file

Additional file 1: The included patient-reported outcome measures (PROMs) in the DiaPROM trial feasibility study. (PDF 123 kb)

## Abbreviations

CGM: Continuous glucose monitoring
EPR: Electronic patient records
FGM: Flash glucose monitoring
IT: Information technology
PAID: Problem Areas in Diabetes scale
PCDS: Perceived Competence in Diabetes Scale
PROMs: Patient-reported outcome measures
RAND-12: The RAND-12 Health Status Inventory
RCT: Randomised controlled trial
SBGM: Self-blood glucose measurement
T1D: Type 1 diabetes
WHO-5: World Health Organization 5-Well-Being Index
WLAN: Wireless local area network
