# Supplementary material for: Electronic capturing of patient-reported outcome measures on a touchscreen computer in clinical diabetes practice (the DiaPROM trial): a feasibility study
Source: Pilot Feasibility Stud. 2019 Feb 20;5:29. doi: 10.1186/s40814-019-0419-4 (PMC6381687; doi:10.1186/s40814-019-0419-4)
Supplement: Supplementary file 1 — The included patient-reported outcome measures (PROMs) in the DiaPROM trial feasibility study. (PDF 123 kb) [file 40814_2019_419_MOESM1_ESM.pdf]

Additional file 1: The included patient-reported outcome measures (PROMs) in the DiaPROM trial feasibility study

PROMs	# items	Scoring	Score	Interpretation	
**Problem Areas in Diabetes scale** (PAID) [11, 27]	20	**5 point Likert scale**<br>0 "not a problem" → 4 "serious problem"<br>Scores are summated. Raw score 0 to 80 is transformed to 0-100 by multiplying by 1.25.	0-100<br><br>Item scores	≥ 40<br>≥ 30<br><br>≥ 3	Serious diabetes-related distress.<br>Moderate diabetes-related distress.<br><br>Somewhat serious to serious distress regarding a particular topic.
**Perceived Competence in Diabetes Scale** (PCDS) [33]	4	**7 point Likert scale**<br>1 "strongly disagree" → 7 "strongly agree"<br>Scores are summated and divided by 4 to form a mean score.	1-7	-	Higher scores indicate better perceived competence.
**Awareness of hypoglycaemia** [34]	1	**Visual analogue scale of 1 to 7**<br>1 "always aware" → 7 "never aware"<br>Item score.	1-7	-	Higher scores indicate lower awareness of hypoglycaemia. A score of ≥4 implies impaired awareness of hypoglycemia.
**High/Low/Varied blood glucose**	3	**Visual analogue scale of 1 to 7**<br>1 "not at all" → 7 "most of the time"<br>Item scores.	1-7	-	Lower scores indicate better perceived control over blood glucose fluctuations.
**WHO-5 Well-being index** (WHO-5) [35]	5	**6 point Likert scale**<br>0 "never" → 5 "all of the time"<br>Scores are summated. Raw score 0 to 25 is transformed to 0-100 by multiplying by 4.	0-100	≤ 50<br><br>< 28	Indication of suboptimal well-being and further testing is recommended.<br>Likely depression.
**RAND-12 Health Status Inventory** (RAND-12) [37, 38]	12	Response options differ.<br>6 items create the physical health component (PHC) and 6 items create the mental health component (MHC).	PHC<br>&<br>MHC	> 50<br>40-49<br>30-39<br>< 30	Indicates person is likely to be well.<br>Indicates person has mild disability.<br>Indicates person has moderate disability.<br>Indicates person has severe disability

Electronic capturing of patient-reported outcome measures on a touchscreen computer in clinical diabetes practice (the DiaPROM trial): a feasibility study.
DOI: 10.1186/s40814-019-0419-4
